# Electrogeneration of a Free-Standing Cytochrome c—Silica Matrix at a Soft Electrified Interface

**DOI:** 10.1021/acs.langmuir.1c00409

**Published:** 2021-03-25

**Authors:** Alonso Gamero-Quijano, Manuel Dossot, Alain Walcarius, Micheál D. Scanlon, Grégoire Herzog

**Affiliations:** † The Bernal Institute and Department of Chemical Sciences, School of Natural Sciences, University of Limerick (UL), Limerick V94 T9PX, Ireland; ‡ Université de Lorraine, CNRS, LCPME, F-54000 Nancy, France

## Abstract

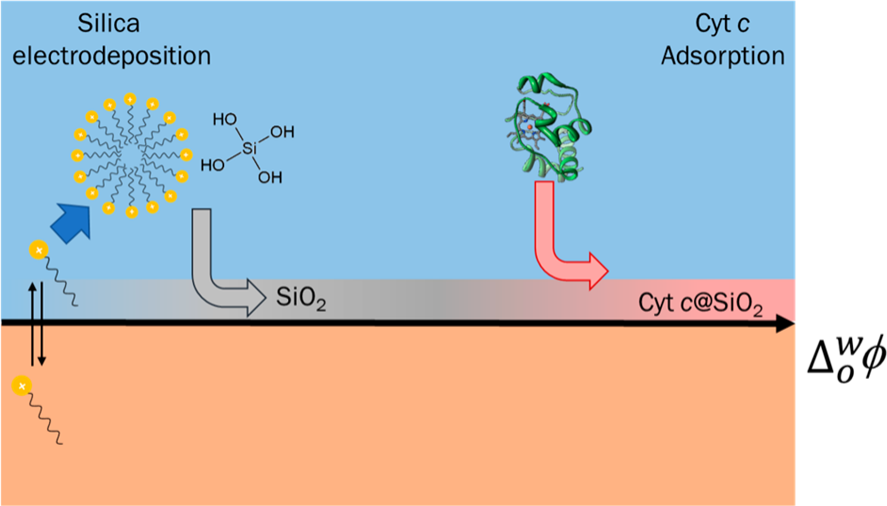

Interactions of a protein with a solid–liquid or a liquid–liquid interface may destabilize its conformation and hence result in a loss of biological activity. We propose here a method for the immobilization of proteins at an electrified liquid–liquid interface. Cytochrome c (Cyt c) is encapsulated in a silica matrix through an electrochemical process at an electrified liquid–liquid interface. Silica condensation is triggered by the interfacial transfer of cationic surfactant, cetyltrimethylammonium, at the lower end of the interfacial potential window. Cyt c is then adsorbed on the previously electrodeposited silica layer, when the interfacial potential, Δ_o_^w^ϕ, is at the positive end of the potential window. By cycling of the potential window back and forth, silica electrodeposition and Cyt c adsorption occur sequentially as demonstrated by *in situ* UV–vis absorbance spectroscopy. After collection from the liquid–liquid interface, the Cyt c–silica matrix is characterized *ex situ* by UV–vis diffuse reflectance spectroscopy, confocal Raman microscopy, and fluorescence microscopy, showing that the protein maintained its tertiary structure during the encapsulation process. The absence of denaturation is further confirmed *in situ* by the absence of electrocatalytic activity toward O_2_ (observed in the case of Cyt c denaturation). This method of protein encapsulation may be used for other proteins (e.g., Fe–S cluster oxidoreductases, copper-containing reductases, pyrroloquinoline quinone-containing enzymes, or flavoproteins) in the development of biphasic bioelectrosynthesis or bioelectrocatalysis applications.

## 1 Introduction

Immobilization of proteins is often sought for applications in the fields of bioanalysis and biocatalysis.^1^ Proteins immobilized onto a substrate offer a more convenient handling, provide a separation from the product, and improve the storage and operational stability over time.^2^ Nevertheless, the control of the protein environment during and after the immobilization process ensures that the immobilized protein will retain its biological activity. Indeed, hydrophobic and electrostatic interactions of a protein at a solid–liquid or liquid–liquid interface may impact the stability of its secondary structure and hence lead to a loss of biological activity.^3^ Furthermore, the direct environment of the protein (*e.g*., pH, ionic strength, temperature, solvent polarity, protein isoelectric point, size, and shape, etc.) can alter the adsorption. In the field of bioelectrocatalysis, various strategies have been envisaged to create a favorable environment to maintain the protein conformation and hence activity.^4^

The behavior of various proteins at polarized liquid–liquid interfaces (a.k.a., interfaces between two immiscible electrolyte solutions, ITIES) has been investigated by electrochemical means.^5,6^ It was shown that proteins such as Cytochrome c (Cyt c),^7−9^ insulin,^10^ hemoglobin,^11,12^ lysozyme,^13^ myoglobin,^14^ albumin,^15^ ferritin,^16^ and thrombin^17^ behaved in a similar manner at the ITIES. Adsorption of the protein at the ITIES was induced by the application of a potential difference more positive than the potential of zero charge (PZC). At the positive end of the potential window, the transfer of the anion of the organic phase background electrolyte was assisted by the adsorbed proteins through hydrophobic and electrostatic interactions with the partially unfolded proteins as demonstrated experimentally^18−20^ and supported by molecular dynamics simulations.^21,22^ In the case of Cyt *c*, partial denaturation of the protein was responsible for a bioelectrocatalytic O_2_ reduction reaction observed at the ITIES.^23^ Preventing protein denaturation at the ITIES remains a challenge. Protein encapsulation within biocompatible silica matrices may solve this issue.^24−32^ For instance, Montilla et al. have shown that Cyt c species present long-term stability if they are encapsulated inside silica matrices, made of a mixture of silanes and methylated silanes.^33,34^ Recently, Poltorak et al. reported the encapsulation of three proteins (hemoglobin, acid phosphatase, and α-amylase) in a silica matrix by a codeposition process at the ITIES under acidic conditions.^35,36^ After encapsulation, these proteins presented interfacial activity by assisting anion transfer from the organic to the aqueous phase. These features are typically observed with macromolecules adsorbed at electrified liquid–liquid interfaces.^37,38^ Thus, the encapsulation within silica networks might provide soft immobilization conditions at the ITIES delaying the denaturation process during the external biasing at positive potentials. Furthermore, it could extend the lifetime of the proteins at the ITIES and facilitate their extraction for further *ex situ* assays.

In the present manuscript, Cyt c was selected as a model redox protein to be encapsulated within silica films formed at the ITIES. Cyt c is a component of the mitochondrial electron transport chain and is heavily involved in the cell death process known as apoptosis.

Herein, we present a novel method of protein encapsulation following a silica sol–gel process. The silica film formation is prompted by the electroassisted ion-transfer of cetyltrimethylammonium (CTA^+^), favoring the silica precursor condensation at the ITIES.^39^ The encapsulation process is carried out under mild conditions to avoid any denaturation induced by hydrophobic interactions with organic anions from the organic phase. We have characterized Cyt c–silica matrix by both spectroscopy (UV–vis absorption spectroscopy, fluorescence, and Raman confocal microscopy) and electrochemical techniques.

## 2 Experimental Section

### 2.1 Reagents

Cyt c from bovine heart ≥95% was purchased from Sigma-Aldrich in the oxidized form (Cyt c-Fe^III^) and used without further purification. The organic phase background electrolytes (bis(triphenylphosphoranylidene)ammonium tetrakis(pentafluorophenyl)borate—BA^+^TB^-^) and cetyltrimethylammonium tetrakis(pentafluorophenyl)borate (CTA^+^TB^–^) were precipitated by mixing equimolar amounts of bis(triphenylphosphoranylidene)ammonium chloride (BA^+^Cl^-^, 97%, Sigma-Aldrich), hexadecyltrimethylammonium bromide (CTAB, 98%, Sigma-Aldrich), and lithium tetrakis(pentafluorophenyl)borate ethyl etherate (Li^+^TB^-^, 98%, Sigma-Aldrich), respectively. Further details regarding the preparation of the organic electrolytes are given in ref (40). Trifluorotoluene (TFT, ≥99%, Sigma-Aldrich) and 1,2 dichloroethane (DCE, ≥99%, Alfa Aesar) were used as organic solvents without further purification. Sodium chloride (≥98%, Prolabo) was used as an aqueous electrolyte for the interfacial silica film formation. The silica precursors for the film formation were tetraethoxysilane (TEOS, 98%, Alfa Aesar) and triethoxymethylsilane (MTEOS, 99%, Sigma-Aldrich). The pH was adjusted with solutions of 1 M HCl (1 M, volumetric solution, Riedel-de Haen) and 1 M NaOH (from pellets, pure, Riedel-de Haen). Ferric chloride hexahydrate (FeCl_3_·6H_2_O, 99–100%, Fluka) was used to prepare silver/silver chloride pseudo-reference electrodes. The electroactivity of encapsulated Cyt c was tested at pH 7 using a phosphate buffer solution (PBS) prepared from phosphate buffer saline tablets purchased from Sigma-Aldrich for a final concentration of 2 mM phosphate buffer, 0.54 mM KCl, and 27.4 mM NaCl. Decamethylferrocene (DcMFc, 97%, Sigma-Aldrich) was used as purchased as the electron donor to assess the electroactivity of the Cyt c@silica films. Purified water (18.2 MΩ cm) was used to prepare all the aqueous solutions, supplied from a Millipore milli-Q water purification system. All other reagents were of the highest grade available and used as received.

### 2.2 Cyt c@SiO_2_ Hydrogel Electrogeneration

**Scheme 1:**
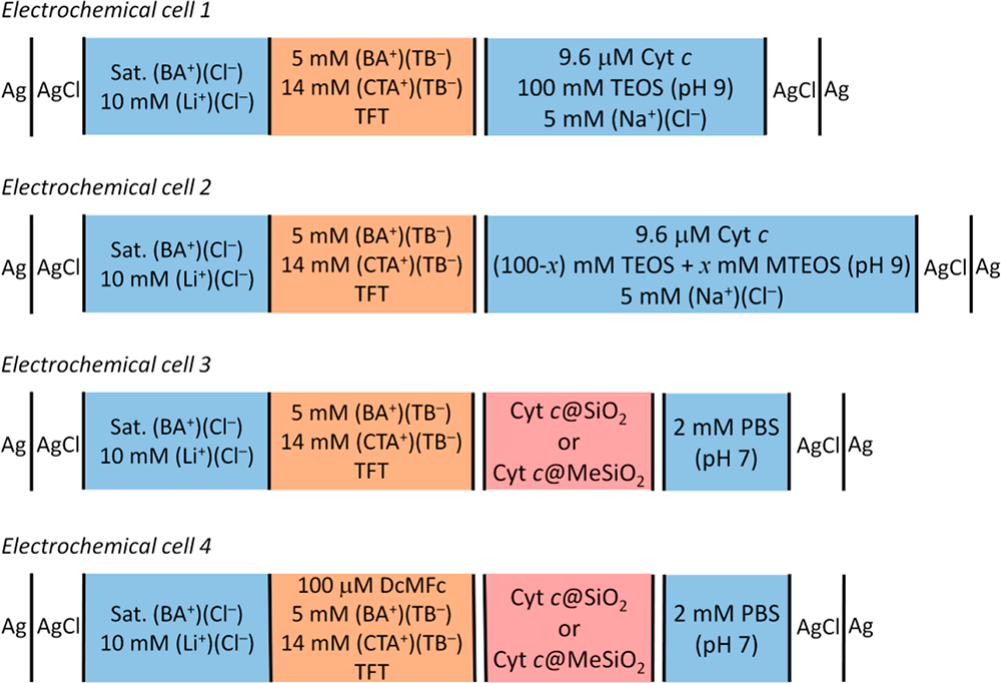
Four-Electrode Electrochemical Cell Configurations Investigated

The sol was prepared as follows: (i) 100 mM of TEOS was hydrolyzed in 10 mL of 5 mM NaCl solution at pH 3 under constant stirring for 3 h (adjusted with HCl), after which the hydrolysis was considered complete; (ii) the pH was then increased to pH 9 (by addition of NaOH), granting the formation of negatively charged silica oligomers; (iii) 3 mg of bovine heart Cyt c was dissolved in 4 mL of the hydrolyzed solution and used as the aqueous phase in electrochemical cells 1 and 2 (see cells 1 and 2 in Scheme 1). In the case of Cyt c@MeSiO_2_, part of the TEOS precursor was replaced by methyltriethoxysilane (MTEOS). The total moles of silica precursor were kept constant (1 mmol). Cyt c@SiO_2_ and Cyt c@MeSiO_2_ hydrogels were electrogenerated at the liquid–liquid interface in a custom-made four-electrode cell with three arms: two Luggin capillaries for the reference electrodes and one arm to add electroactive species into the organic phase (Figure S1). The geometrical area of the interface was 1.53 cm^2^.

The interfacial potential difference, Δ_o_^w^ϕ, was controlled with a PINE wavedriver 20 potentiostat (Pine research, USA). Cyt c@SiO_2_ or Cyt c@MeSiO_2_ hydrogels were electrogenerated by cyclic voltammetry at a slow scan rate (1 or 2 mV s^–1^). The thickness of the silica deposits can be controlled by the number of scans performed at the ITIES. Here, the number of scans was varied from two to seven. For electron transfer experiments, a silica thin film was deposited by performing two voltammetric scans. For ex situ characterization, a thicker film was needed; therefore, the number of voltammetric scans was increased to seven. Once formed, the hydrogels were prepared for further characterization. For *ex situ* Raman and reflective UV–vis spectroscopy, the hydrogel was collected with a spatula and rinsed with a solution of v:v 1:10 ethanol:1 mM HCl by immersion for 2 h. This allowed the removal of the organic electrolyte and CTA^+^ template. Next, the hydrogels were rinsed with a mixture of v:v 1:10 acetone:distilled water and dried overnight in the oven at 40 °C. For the *in situ* electrochemical characterization, the aqueous phase used for the electrogeneration was carefully removed from the electrochemical cell and replaced with 2 mM PBS solution. This process was repeated several times before achieving electrochemical stabilization of the film in the new aqueous phase electrolyte solution cyclic voltammetry through 20 repetitive scans at 20 mV s^–1^; see cell 3 in Scheme 1.

### 2.3 Cyt c@SiO_2_ and Cyt c@MeSiO_2_ Hydrogel Characterization

*In situ* UV–vis experiments were carried out with a parallel beam configuration using a USB 2000 Fiber Optic Spectrometer (Ocean Optics, USA). The light beam was generated using a DH-2000-BAL deuterium–halogen light source (Ocean Optics, USA) and guided through the optical fiber of 600 μm of diameter (Ocean Optics, USA). The light beam was collimated using optical lenses (Thorlabs, focal length: 2 cm) before and after the transmission of the beam through the electrochemical cell (Figure SI2). The potential was controlled using an Autolab PGSTAT204 potentiostat (Metrohm, Switzerland). Confocal Raman spectroscopy measurements were carried out using a WITec 300R spectrometer with a green light laser (532 nm) as excitation source, equipped with a polarizing beam splitter for polarized light experiments. The samples were placed on a glass slide and mounted in the focal plane of an Olympus X50 objective. The laser spot was around 2 μm^2^. Raman mappings were analyzed using WITec project 2.08 software. UV–vis diffuse reflectance spectroscopy (UV–vis DRS) was recorded using a UV–visible–NIR spectrometer (Cary 6000, Agilent), and the preparation of the sample (pellets) was 5 mg of sample mixed with 95 mg of KBr. Fluorescence microscopy was performed using an Olympus BX3-URA fluorescence microscope with a mercury lamp as the excitation source and a Power Supply Unit U-RFL-T. The samples were excited at 360 nm, and the fluorescence images were acquired using a 420 nm filter. Interfacial electron transfer reactions at the liquid–liquid interface were investigated after the addition of an aliquot of DcMFc to the organic phase. Five hundred microliters of a 1 mM DcMFc solution was added carefully through the third arm of the homemade four-electrode cell; see cell 4 in Scheme 1. The organic solution was stirred for 3 min using a PTFE magnetic bar; further details about the electrochemical setup are shown in Figure S1B.

## 3 Results and Discussion

### 3.1 Encapsulation of Cytochrome c within Silica Matrices by Electrochemistry at the ITIES

**Figure 1:**
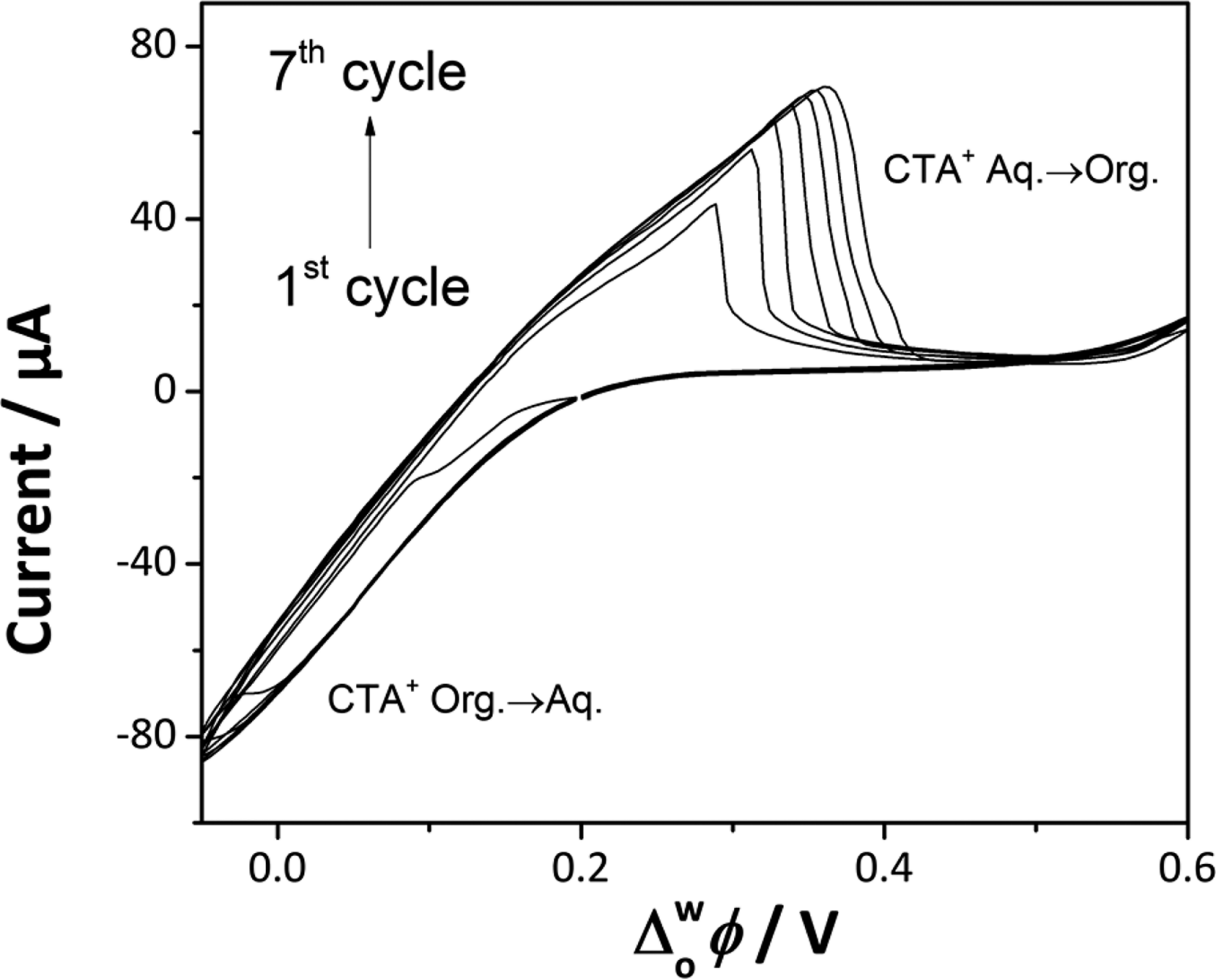
Cyclic voltammograms recorded at the liquid–liquid interface during the Cyt c@SiO_2_ films formation using electrochemical cell 1 (see Scheme 1) at a scan rate of 1 mV s^–1^.

Cyt c@silica films at the ITIES were obtained by cyclic voltammetry. The interfacial transfer of CTA^+^ (a cationic surfactant) triggered the condensation of hydrolyzed TEOS molecules^39−41^ (Figure 1) and Cyt c encapsulation. The potential was scanned at 1 mV s^–1^ from the open-circuit potential (Δ*E* = +0.19 V) toward the negative end of the potential window. The first cycle showed a sharp negative current appearing between +0.10 and −0.05 V. This current was attributed to the transfer of CTA^+^ from the organic to the aqueous phase.^40^ Here, the CTA^+^ transfer was facilitated by the presence of negatively charged siloxane oligomeric species, which are abundant at pH 9.^42^ Once the CTA^+^ was transferred to the aqueous phase, these ions led to the formation of charged micelles, which were surrounded by the silica species, accelerating their condensation, and thereby a hydrogel was formed. Upon reversing the scan toward more positive potentials, a peak attributed to a partial back-transfer of CTA^+^ appeared at +0.28 V.^43^ Further changes in polarization toward more positive potentials have shown a capacitive current attributed to the double layer of the liquid–liquid interface. Repetitive scans have shown a constant increase in the peak intensity attributed to the CTA^+^ back-transfer, which indicated the thickening of SiO_2_ deposits at the liquid–liquid interface. Control experiments demonstrated that the formation of the silica deposits was not spontaneous; cell 1 was left for about 1 h at an open-circuit potential without the formation of a silica film at the liquid–liquid interface. It is worth mentioning that the electrochemical response of Cyt c at pH 9 is featureless within the potential window range used for the Cyt c@silica films syntheses (−0.05 to 0.60 V). Therefore, all the voltammetric features shown in Figure 1 should be attributed to the silica film formation. As a control, the electrochemical behavior of Cyt c in the absence of CTA^+^ and TEOS is shown in Figure SI3. Cyt c adsorption is observed at the positive end of the potential window around +0.90 V, which is agreement with previous studies.^7−9^

**Figure 2:**
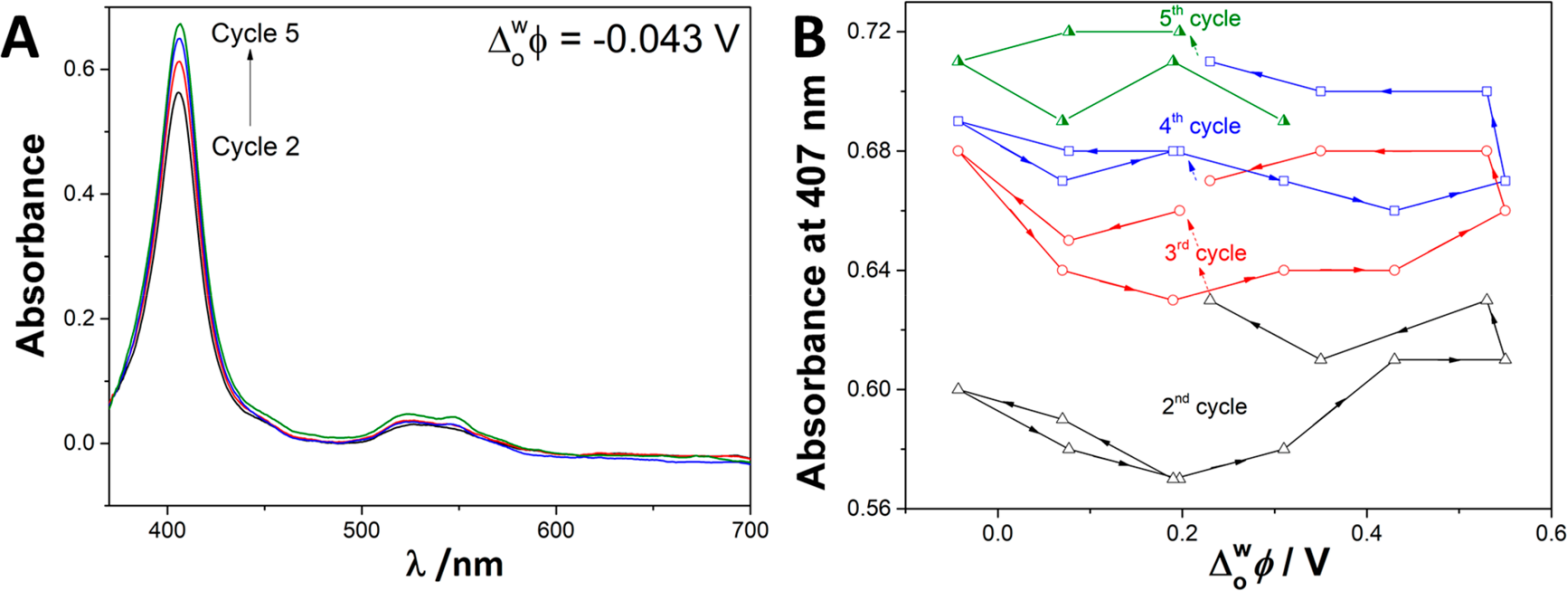
(A) *In situ* UV–vis absorption spectra of Cyt c at −0.04 V and (B) variation of the absorbance at 407 nm during the cyclic voltammetry cycling experiments performed in Figure 1, using electrochemical cell 1 (see Scheme 1) at a scan rate of 1 mV s^–1^.

Studies on the immobilization of proteins within nanoporous supports have shown that the efficiency of immobilization was conditioned by a combination of electrostatic interactions, ionic strength, nature of counterions, and van der Waals forces.^44−46^ Herein, the pH of the aqueous phase was lower than the Cyt c isoelectric point (pI 9.8).^47,48^ Thus, our conditions of negatively charged silica film formation should favor the encapsulation of positively charged Cyt c. In order to confirm our hypothesis, the silica sol–gel film formation was followed by *in situ* parallel beam UV–vis absorbance spectroscopy (Figure 2). Here, the beam was parallel to the aqueous side of the liquid–liquid interface where Cyt c and the silica precursor were dissolved. Each absorbance spectrum was recorded at −0.04 V. The experimental setup allowed the recording of the UV–vis absorption spectra of Cyt c located near the interface during the silica film condensation.

The electronic spectrum of Cyt c presents two characteristic bands: (i) the Soret band centered at 407 nm and (ii) the Q-band centered at 533 nm. Both bands are attributed to the absorption of the porphyrin chromophore of Cyt c (see Figure 2A). The Soret band is attributed to π–π* transitions in the porphyrin ring structure of the Cyt c-Fe^III^ (heme center) and is an indicator of the native-like state of the protein. A blue shift of the Soret band is commonly attributed to protein denaturation, whereas a red shift is related to a change in the protein redox state.^34,49−51^ The Q-band between 500 and 565 nm showed two absorption bands known as α- and β-bands, which are poorly defined when the Cyt c is in an oxidized state (Fe^III^).^52,53^ The absorbance of the Soret and Q-bands increased after each cycle, suggesting that the molar concentration of Cyt c near the interface increased after each voltammetric scan. The Soret band was centered at 407 nm and was constant throughout the silica film formation thoroughly suggesting that Cyt c encapsulation occurred without denaturation or changes in the redox state (Figure 2A).

Figure 2B shows the absorbance of the Soret band vs the potential for each voltammetric cycle. The potential sweep started from +0.20 V toward less positive potentials. The Soret band intensity increased during the second cycle (red line), from +0.20 toward −0.05 V, confirming that an accumulation of Cyt c species occurs in the vicinity of the liquid–liquid interface during negative biasing.^40^ When the potential sweep was reversed from −0.05 toward +0.20 V, the intensity of the Soret band decreased, returning to its initial value observed at +0.20 V (Figure 2B). This suggested that partial desorption of Cyt c occurred. Another increase of the Soret band absorbance was observed in the potential region from +0.25 to +0.60 V; this was attributed to the presence of positive aqueous species and organic anions (TB^–^) at the liquid–liquid interface that is favored at positive potentials (Figure SI3). Therefore, a positive biasing favored the accumulation of Cyt c species on the aqueous side of the interface, suggesting a second accumulation step. The reverse scan from +0.60 toward +0.20 V did not show significant changes in the Soret band absorbance, indicating that the concentration of Cyt c was constant in the vicinity of the interface. The subsequent cycles presented similar features as the second one. Overall, i*n situ* parallel beam UV–vis experiments suggested that Cyt c encapsulation should occur at two different stages of interfacial polarization: (i) at negative potentials while the silica sol–gel formation is formed and (ii) at positive potentials due to electrostatic and hydrophobic interactions with preformed silica deposits and TB^–^ species from the organic phase, respectively.

### 3.2 Spectroscopic Characterization of Cyt c@SiO_2_ Hydrogels

**Figure 3:**
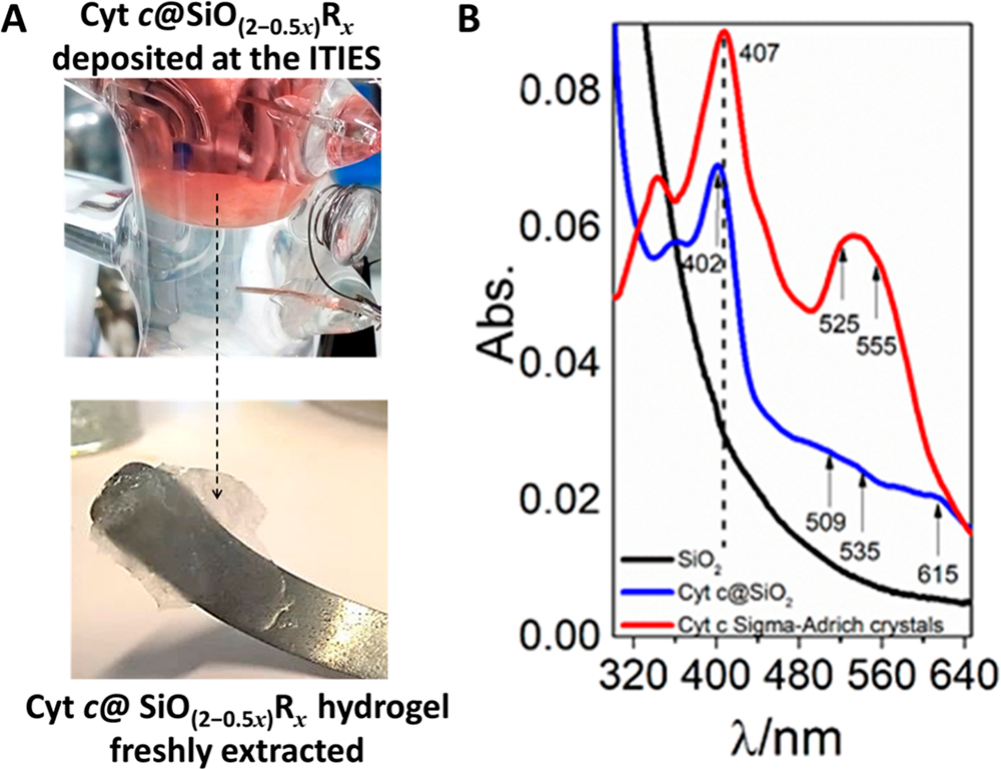
(A) Top: Photograph of the Cyt c@SiO_2_ films deposited at the interface after seven repetitive scans at 1 mV s^–1^; bottom: Cyt c@SiO_2_ film collected at the liquid–liquid interface. (B) UV–vis DRS of pure Cyt c powder (red curve), Cyt c@SiO_2_ films after CTA^+^ extraction (blue curve), and SiO_2_ deposits in the absence of Cyt c (black curve).

A robust and slightly reddish hydrogel, formed after seven voltammetric cycles (Figure 3A), was collected from the interface, and these Cyt c@SiO_2_ hydrogel films were characterized by UV–vis DRS and Raman spectroscopy. Further details about sample treatment are described *vide supra* (see Sections 2.2 and 2.3). Figure 3B shows the UV–vis spectra of Cyt c crystals, Cyt c-free silica, and Cyt c@SiO_2_ films. The UV–vis spectrum of Cyt c crystals presented a Soret band centered at 407 nm and a well-defined Q-band (red line). The UV–vis spectrum of encapsulated Cyt c (blue line) presented a Soret band centered at 402 nm, and the absorbance of the Q-band decreased with the appearance of new peaks centered at 509 and 535 nm. The variations of the Q-band absorption and the appearance of new Q-band peaks at 509 and 535 nm indicated that the low-spin hemes of Cyt c were converted to the high-spin form.^54,55^ This was confirmed by the appearance of a new band centered at 615 nm which was attributed to in-plane charge transfer between the porphyrin and heme iron of the high-spin species.^56−58^ The change from the low-spin (native Cyt c) to the high-spin configuration indicates that the sixth ligand of heme is no longer the Met-80 residue and has been replaced by a different residue or by water molecules. However, encapsulated Cyt c still retained its integrity after the immobilization, which was confirmed by the retention of well-defined Soret band features. Denatured Cyt c would have shown a significant decrease in absorbance, a substantial blue shift, and a broadening of the Soret band.^47,57,59^ Note that a blue shift of ca. 5 nm of the Soret band and the decrease of the Q-band intensity could indicate changes in the microenvironment of the encapsulated protein caused by the loss of the water after the drying process.^27,60^ Indeed, a decay of the Q-band might be related to strong electrostatic interactions within the silica pores.^61^ The UV–vis diffuse reflectance spectrum of pure silica deposits (Figure 3B, black curve) did not show any features at 407 nm or in the 500–600 nm region. Therefore, the spectrum taken for the encapsulated Cyt c must be purely attributed to electronic transitions of the heme group.

**Figure 4:**
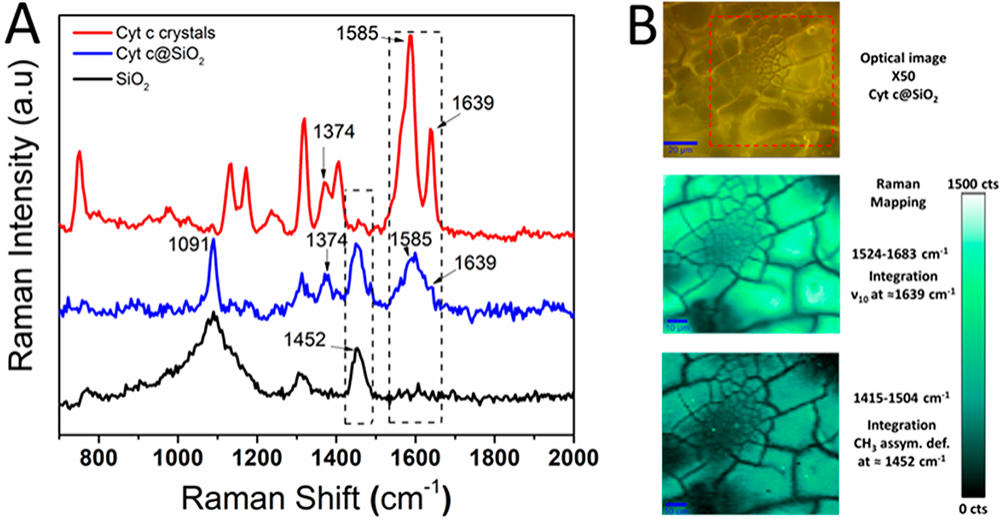
(A) Raman spectra of Cyt c crystals (red curve), Cyt c@SiO_2_ (blue curve), and SiO_2_ deposit (black curve). (B) Optical image of Cyt c@SiO_2_ (top) and Raman mapping considering a band specific to Cyt c (ν_10_ at 1639 cm^–1^, middle) and a band specific to SiO_2_ (CH_3_ asymmetric deformation of partially hydrolyzed TEOS at 1452 cm^–1^, bottom) as the integration peaks, respectively. Scale bar is 20 μm for the optical image and 10 μm for the Raman mapping.

UV–vis DRS studies have shown that Cyt c species were successfully immobilized within the silica films; however, the distribution of the protein within the silica matrix was still uncertain. Therefore, Raman scattering measurements were performed to study the distribution and the structural changes of Cyt c within the silica films.^62−65^ Most of Raman vibrational modes of Cyt c have been described in detail by Spiro et al.^62,63,66^ and assigned to active vibrational modes of the heme group (π → π* transition in the porphyrin ring). Figure 4A shows the Raman spectra of Cyt c crystals (red line), Cyt c@SiO_2_ (blue line), and SiO_2_ (black line) films. The vibrational studies were performed in the range of 1000–1650 cm^–1^ since most of the relevant Raman modes belong to this vibrational window.

The Raman spectrum of Cyt c crystals (red line) showed vibrational modes at 1585 and 1639 cm^–1^ known as ν_2_ and ν_10_, respectively, corresponding to Raman modes of the amide-I symmetric stretching (C_β_–C_β_) and (C_α_–C_m_).^65^ Here, the band ν_2_ is considered as a spin-state marker, whereas ν_10_ is considered a band sensitive to structural changes in the protein.^67^ Thus, the Cyt c crystals were oxidized and in their native state (Cyt c Fe^III^). It is worth mentioning that any down-shift of the ν_4_ and ν_10_ bands could be attributed to changes in the oxidation state or in the structural conformation of Cyt c, respectively.

The Raman spectrum of a Cyt c@SiO_2_ film (blue line) revealed well-defined ν_4_ and ν_10_ modes. These bands did not overlap with the SiO_2_ stretching bands (see black line). The appearance of a broad band centered at ca. 1596 cm^–1^ indicated the presence of a mixed tertiary structure and intermingled α-helical and β-sheet secondary structures.^65^ These conformational changes were attributed to the strong interactions between the silica walls and the Cyt c. With regards to the ν_4_ mode centered at 1374 cm^–1^, this band did not show any shift for the Cyt c@SiO_2_ film indicating that the encapsulated protein kept its initial oxidation state. The Raman spectrum for the Cyt c-free silica film (black line) has shown bands centered at 1091 and 1452 cm^–1^; these bands were attributed to C–O asymmetric stretching and CH_3_ asymmetric deformation of partially hydrolyzed TEOS, respectively.^68^

The primary concern during the encapsulation process was Cyt c agglomeration within the silica matrix. Therefore, Raman mapping was used to verify the even distribution of Cyt c at the microscopic level (Figure 4B). To do so, we have selected one Raman band specific to Cyt c and another one for SiO_2_. We used the broad band centered at 1596 cm^–1^, which included the pair of peaks centered at 1585 and 1639 cm^–1^ attributed to the vibration modes ν_2_ and ν_10_ of Cyt c. We carried out the integration of the band centered at 1452 cm^–1^ to identify the SiO_2_-rich region of the sample. The mapping of Cyt c@SiO_2_ pointed out that both SiO_2_ and Cyt c were evenly distributed within the sample, suggesting an uniform distribution of Cyt c within the silica during the silica deposition process.

**Figure 5:**
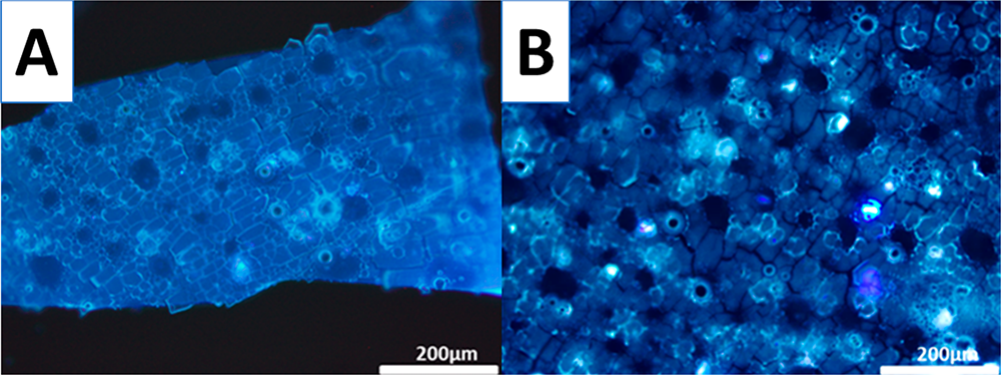
Fluorescence micrographs of Cyt c@SiO_2_ deposits before (A) and after (B) 0.1 M HCl:ethanol treatment.

Experiments of fluorescence microscopy confirmed the results obtained by Raman spectroscopy suggesting the presence of a mixed tertiary structure of Cyt c species within the silica film (Figure 5). Tryptophan fluorescence is a convenient method to investigate the local conformation of a protein.^69,70^ Tryptophan-59 of Cyt c forms a hydrogen bond to one of the propionic groups of the heme center. In the native state, the fluorescence emission intensity of this group is low due to quenching of the emission signal.^71−73^ However, a partial unfolding of the protein decreases the fluorescence quenching, and the emission intensity of Tryptophan-59 became measurable.^71^ The fluorescent emission micrograph did not show fluorescent regions, suggesting that Cyt c was encapsulated in a nativelike state, as suggested by UV–vis DRS and Raman studies (Figure 5A). The Cyt c@SiO_2_ film was then treated using a mixture v:v 1:1 of 0.1 M HCl:ethanol for 2 h under constant stirring to chemically induce denaturing of the encapsulated protein. After application of this chemical treatment, the appearance of the film was not affected. However, the emission intensity increased noticeably all over the film (Figure 5B), with several fluorescent hot spots attributed to the unfolded structure of Cyt c observed. The silica film provided a protective environment to the protein as immersion of the Cyt c@SiO_2_ film in a 0.4 M NaCl solution for 2 h did not show any signs of denaturation (Figure S5).

Based on the *ex situ* spectroscopic characterization of the Cyt c@SiO_2_ hydrogel, Cyt c retained its tertiary structure and did not unfold during the encapsulation process at the ITIES. This result suggests that other proteins (enzymes, monoclonal antibodies, among others) could be immobilized and studied at the interface following a similar *in situ* sol–gel encapsulation process at the ITIES.

### 3.3 Electrochemical Characterization of Cyt c@SiO_2_ Hydrogels

In our recent breakthrough study we have shown that, in the absence of silica, Cyt c adsorbs on the interface by the application of an interfacial potential difference higher than the PZC.^23^ Under the positive polarization of the interface, Cyt c is oriented with the active heme group facing toward the organic phase, where it is accessible for an electron transfer reaction with the organic reductant decamethylferrocene (DcMFc). Cyclic voltammetry in the presence of Cyt c and DcMFc showed two processes: (i) a rise of current at an onset potential at +0.50 V and (ii) a reversible ion transfer at a half-wave potential at +0.20 V (Figure S4). The first process was attributed to the interfacial electron transfer reaction between Cyt c and DcMFc, while the second process was linked to the transfer of the DcMFc^+^ cations generated. The interfacial electron transfer is the consequence of O_2_ catalytic reduction by Cyt c partially denatured by interfacial adsorption and its interaction with organic TB^–^. In its native state, Cyt c does not catalyze O_2_ reduction, but upon partial denaturation by polarization of the ITIES, Cyt c becomes a potential O_2_ reduction catalyst.^23^

**Figure 6:**
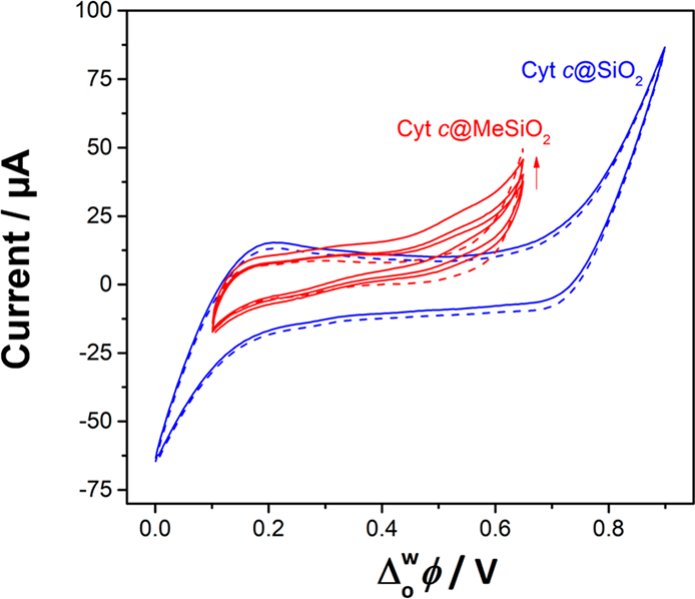
Cyclic voltammograms of Cyt c@SiO_2_ (blue curve) and Cyt c@MeSiO_2_ (red curves) modified ITIES in the absence (dashed curves) and in the presence (solid curves) of 100 μM DcMFc in the organic phase. They cyclic voltammetry was performed using electrochemical cell 4 (see Scheme 1) at a scan rate of 20 mV s^–1^. The arrow represents the current evolution with the repetition of scans in the case of Cyt c@MeSiO_2_.

*Ex situ* spectroscopic characterization, reported in Section 3.2, suggested that Cyt c encapsulated in silica (Cyt c@SiO_2_) retained its native conformation and hence should not catalyze O_2_ reduction. A Cyt c@SiO_2_ hydrogel was thus prepared by cyclic voltammetry using electrochemical cell 2. Once the Cyt c@SiO_2_ was formed, the aqueous phase was carefully replaced with a phosphate buffer solution at pH 7 (Figure S6). The silica film remained intact after the replacement of the aqueous solution. Indeed, it did not show macroscopic defects; it was flexible and robust, with waves or curls at the interface suggesting a membranelike behavior. A stable cyclic voltammetry signal was obtained after 20 repetitive cycles. The stabilized CV presented a featureless blank CV with a typical ion transfer of background electrolyte at both negative and positive end potentials (Figure S6). After the addition of 100 μM of DcMFc to the organic phase, no electrocatalytic activity toward O_2_ reduction was observed, confirming that Cyt c kept its native conformation during the encapsulation (Figure 6). This may be ascribed to electrostatic interactions between Cyt c and the surface of the silica pores,^34^ avoiding further hydrophobic interactions and partial protein denaturation.

Since the environment nearby a protein plays a major role in its electroactivity,^33,34,74^ Cyt c species were encapsulated within organic–inorganic hybrid silica films. A hybrid Cyt c@silica film was prepared from a sol which contained 10% of methyltriethoxysilane (MTEOS). These Cyt c@MeSiO_2_ films presented an interfacial electroactivity in the presence of DcMFc with an onset of current at +0.45 V, attributed to O_2_ reduction (Figure 6). The interfacial electroactivity observed with hybrid Cyt c@MeSiO_2_ films might be due to the higher hydrophobicity,^75^ allowing the organic phase to diffuse through the hybrid silica gel and thus causing partial denaturation of the encapsulated Cyt c. These experiments show that the control of Cyt c’s immediate environment can protect the biomolecule from denaturation^26^ despite the application of a positive interfacial potential difference and the close proximity of the organic phase.

## 4 Conclusions

This work demonstrated that Cyt c can effectively be encapsulated in a silica hydrogel during the cycling of the potential window at the ITIES due to subsequent silica condensation and Cyt c adsorption. This two-step electrochemical process was followed by *in situ* UV–vis absorption spectroscopy. After the hydrogel formation, *ex situ* spectroscopic characterization (confocal Raman microscopy, fluorescence spectroscopy, and UV–vis diffusive reflectance spectroscopy) showed that Cyt c was deposited in its native state. This was also confirmed by the absence of O_2_ catalyzed by denatured Cyt c at the ITIES. This method for the encapsulation of Cyt c at soft polarized interfaces could be used for other types of proteins in the field of bioelectrosynthesis or bioelectrocatalysis, where the low water solubility of hydrophobic substrates or products may limit the conversion rate of chiral compounds of pharmaceutical interest.^76^

## Supporting Information Available

Experimental setups for electrochemistry and spectroelectrochemistry measurements; control cyclic voltammetry of Cyt c in the absence of silica hydrogel; protective effect of the silica; electrochemical stabilization of Cyt c@SiO_2_ hydrogel after aqueous phase exchange (PDF)

## References

[ref1] Gray C. J.; Weissenborn M. J.; Eyers C. E.; Flitsch S. L. Enzymatic Reactions on Immobilised Substrates. Chem. Soc. Rev. 2013, 42, 6378–6405. 10.1039/c3cs60018a. 23579870

[ref2] Sheldon R. A.; van Pelt S. Enzyme Immobilisation in Biocatalysis: Why, What and How. Chem. Soc. Rev. 2013, 42, 6223–6235. 10.1039/C3CS60075K. 23532151

[ref3] Pinholt C.; Hartvig R. A.; Medlicott N. J.; Jorgensen L. The Importance of Interfaces in Protein Drug Delivery – Why Is Protein Adsorption of Interest in Pharmaceutical Formulations?. Expert Opin. Drug Delivery 2011, 8, 949–964. 10.1517/17425247.2011.577062. 21557707

[ref4] Chen H.; Simoska O.; Lim K.; Grattieri M.; Yuan M.; Dong F.; Lee Y. S.; Beaver K.; Weliwatte S.; Gaffney E. M.; Minteer S. D. Fundamentals, Applications, and Future Directions of Bioelectrocatalysis. Chem. Rev. 2020, 120, 12903 10.1021/acs.chemrev.0c00472. 33050699

[ref5] Arrigan D. W. M. Voltammetry of Proteins at Liquid-Liquid Interfaces. Annu. Rep. Prog. Chem., Sect. C: Phys. Chem. 2013, 109, 167–188. 10.1039/c3pc90007j.

[ref6] Arrigan D. W. M.; Hackett M. J.; Mancera R. L. Electrochemistry of Proteins at the Interface between Two Immiscible Electrolyte Solutions. Current Opinion in Electrochemistry 2018, 12, 27–32. 10.1016/j.coelec.2018.07.012.

[ref7] Shinshi M.; Sugihara T.; Osakai T.; Goto M. Electrochemical Extraction of Proteins by Reverse Micelle Formation. Langmuir 2006, 22, 5937–5944. 10.1021/la060858n. 16768533

[ref8] Shinshi M.; Sugihara T.; Osakai T.; Goto M. Erratum: Electrochemical Extraction of Proteins by Reverse Micelle Formation (Langmuir (2006) 22 (5937–5944)). Langmuir 2006, 22, 8614 10.1021/la062188r. 16768533

[ref9] Alvarez de Eulate E.; O’Sullivan S.; Arrigan D. W. M. Electrochemically Induced Formation of Cytochrome c Oligomers at Soft Interfaces. ChemElectroChem 2017, 4, 898–904. 10.1002/celc.201600851.

[ref10] Kivlehan F.; Lanyon Y. H.; Arrigan D. W. M. Electrochemical Study of Insulin at the Polarized Liquid-Liquid Interface. Langmuir 2008, 24, 9876–9882. 10.1021/la800842f. 18666786

[ref11] Herzog G.; Kam V.; Arrigan D. W. M. Electrochemical Behaviour of Haemoglobin at the Liquid/Liquid Interface. Electrochim. Acta 2008, 53, 7204–7209. 10.1016/j.electacta.2008.04.072.

[ref12] Herzog G.; Moujahid W.; Strutwolf J.; Arrigan D. W. M. Interactions of Proteins with Small Ionised Molecules: Electrochemical Adsorption and Facilitated Ion Transfer Voltammetry of Haemoglobin at the Liquid/Liquid Interface. Analyst 2009, 134, 1608–1613. 10.1039/b905441n. 20448927

[ref13] Scanlon M. D.; Jennings E.; Arrigan D. W. M. Electrochemical Behaviour of Hen-Egg-White Lysozyme at the Polarised Water/1,2-Dichloroethane Interface. Phys. Chem. Chem. Phys. 2009, 11, 2272–2280. 10.1039/b815589e. 19305901

[ref14] O’Sullivan S.; Arrigan D. W. M. Electrochemical Behaviour of Myoglobin at an Array of Microscopic Liquid-Liquid Interfaces. Electrochim. Acta 2012, 77, 71–76. 10.1016/j.electacta.2012.05.070.

[ref15] Matsui R.; Sakaki T.; Osakai T. Label-Free Amperometric Detection of Albumin with an Oil/Water-Type Flow Cell for Urine Protein Analysis. Electroanalysis 2012, 24, 1164–1169. 10.1002/elan.201200048.

[ref16] Sakae H.; Toda Y.; Yokoyama T. Electrochemical Behavior of Ferritin at the Polarized Water|1,2-Dichloroethane Interface. Electrochem. Commun. 2018, 90, 83–86. 10.1016/j.elecom.2018.04.010.

[ref17] Felisilda B. M. B.; Arrigan D. W. M. Electroactivity of Aptamer at Soft Microinterface Arrays. Anal. Chem. 2018, 90, 8470–8477. 10.1021/acs.analchem.8b01172. 29893124

[ref18] Hartvig R. A.; Méndez M. A.; van de Weert M.; Jorgensen L.; Østergaard J.; Girault H. H.; Jensen H. Interfacial Complexes between a Protein and Lipophilic Ions at an Oil-Water Interface. Anal. Chem. 2010, 82, 7699–7705. 10.1021/ac101528r. 20735009

[ref19] Alvarez de Eulate E.; Qiao L.; Scanlon M. D.; Girault H. H.; Arrigan D. W. M. Fingerprinting the Tertiary Structure of Electroadsorbed Lysozyme at Soft Interfaces by Electrostatic Spray Ionization Mass Spectrometry. Chem. Commun. 2014, 50, 11829–11832. 10.1039/C4CC05545D. 25156670

[ref20] Booth S. G.; Felisilda B. M. B.; Alvarez De Eulate E.; Gustafsson O. J. R.; Arooj M.; Mancera R. L.; Dryfe R. A. W.; Hackett M. J.; Arrigan D. W. M. Secondary Structural Changes in Proteins as a Result of Electroadsorption at Aqueous-Organogel Interfaces. Langmuir 2019, 35, 5821–5829. 10.1021/acs.langmuir.8b04227. 30955327

[ref21] Arooj M.; Gandhi N. S.; Kreck C. A.; Arrigan D. W. M.; Mancera R. L. Adsorption and Unfolding of Lysozyme at a Polarized Aqueous-Organic Liquid Interface. J. Phys. Chem. B 2016, 120, 3100–3112. 10.1021/acs.jpcb.6b00536. 26950406

[ref22] Arooj M.; Arrigan D. W. M.; Mancera R. L. Characterization of Protein-Facilitated Ion-Transfer Mechanism at a Polarized Aqueous/Organic Interface. J. Phys. Chem. B 2019, 123, 7436–7444. 10.1021/acs.jpcb.9b04746. 31379167

[ref23] Gamero-Quijano A.; Bhattacharya S.; Cazade P. A.; Molina-Osorio A. F.; Beecher C.; Djeghader A.; Soulimane T.; Dossot M.; Thompson D.; Herzog G.; Scanlon M. D. Modulating the Pro-Apoptotic Activity of Cytochrome c at a Biomimetic Electrified Interface. 2021, Preprint, doi.org/10.26434/chemrxiv.14229605.v1. 10.1126/sciadv.abg4119 PMC8570605 34739310

[ref24] Ellerby L. M.; Nishida C. R.; Nishida F.; Yamanaka S. A.; Dunn B.; Valentine J. S.; Zink J. I. Encapsulation of Proteins in Transparent Porous Silicate Glasses Prepared by the Sol-Gel Method. Science 1992, 255, 1113–1115. 10.1126/science.1312257. 1312257

[ref25] Miller J. M.; Dunn B.; Valentine J. S.; Zink J. I. Synthesis Conditions for Encapsulating Cytochrome c and Catalase in SiO2 Sol-Gel Materials. J. Non-Cryst. Solids 1996, 202, 279–289. 10.1016/0022-3093(96)00191-3.

[ref26] Gill I.; Ballesteros A. Encapsulation of Biologicals within Silicate, Siloxane, and Hybrid Sol- Gel Polymers: An Efficient and Generic Approach. J. Am. Chem. Soc. 1998, 120, 8587–8598. 10.1021/ja9814568.

[ref27] Lan E. H.; Dave B. C.; Fukuto J. M.; Dunn B.; Zink J. I.; Valentine J. S. Synthesis of Sol-Gel Encapsulated Heme Proteins with Chemical Sensing Properties. J. Mater. Chem. 1999, 9, 45–53. 10.1039/a805541f.

[ref28] Xu J. S.; Zhao G. C. Direct Electrochemistry of Cytochrome c on a Silica Sol-Gel Film Modified Electrode. Electroanalysis 2008, 20, 1200–1203. 10.1002/elan.200704164.

[ref29] Harper-Leatherman A. S.; Iftikhar M.; Ndoi A.; Scappaticci S. J.; Lisi G. P.; Buzard K. L.; Garvey E. M. Simplified Procedure for Encapsulating Cytochrome c in Silica Aerogel Nanoarchitectures While Retaining Gas-Phase Bioactivity. Langmuir 2012, 28, 14756–14765. 10.1021/la3011025. 22924640

[ref30] Burgos M. I.; Ochoa A.; Perillo M. A. β-Sheet to α-Helix Conversion and Thermal Stability of β-Galactosidase Encapsulated in a Nanoporous Silica Gel. Biochem. Biophys. Res. Commun. 2019, 508, 270–274. 10.1016/j.bbrc.2018.11.077. 30497782

[ref31] Nguyen L.; Döblinger M.; Liedl T.; Heuer-Jungemann A. DNA-Origami-Templated Silica Growth by Sol–Gel Chemistry. Angew. Chem., Int. Ed. 2019, 58, 912–916. 10.1002/anie.201811323. 30398705

[ref32] Catauro M.; Ciprioti S. V. Sol-Gel Synthesis and Characterizationof Hybrid Materials for Biomedical Applications; Springer: Singapore, 2019. 10.1007/978-981-13-0989-2.

[ref33] López-Bernabeu S.; Gamero-Quijano A.; Huerta F.; Morallón E.; Montilla F. Enhancement of the Direct Electron Transfer to Encapsulated Cytochrome c by Electrochemical Functionalization with a Conducting Polymer. J. Electroanal. Chem. 2017, 793, 34–40. 10.1016/j.jelechem.2016.12.044.

[ref34] Gamero-Quijano A.; Huerta F.; Morallón E.; Montilla F. Modulation of the Silica Sol-Gel Composition for the Promotion of Direct Electron Transfer to Encapsulated Cytochrome C. Langmuir 2014, 30, 10531–10538. 10.1021/la5023517. 25111076

[ref35] Poltorak L.; van der Meijden N.; Oonk S.; Sudhölter E. J. R.; de Puit M. Acid Phosphatase Behaviour at an Electrified Soft Junction and Its Interfacial Co-Deposition with Silica. Electrochem. Commun. 2018, 94, 27–30. 10.1016/j.elecom.2018.07.022.

[ref36] Poltorak L.; van der Meijden N.; Skrzypek S.; Sudhölter E. J. R.; de Puit M. Co-Deposition of Silica and Proteins at the Interface between Two Immiscible Electrolyte Solutions. Bioelectrochemistry 2020, 134, 107529 10.1016/j.bioelechem.2020.107529. 32311664

[ref37] Herzog G.; Eichelmann-Daly P.; Arrigan D. W. M. Electrochemical Behaviour of Denatured Haemoglobin at the Liquid|liquid Interface. Electrochem. Commun. 2010, 12, 335–337. 10.1016/j.elecom.2009.12.020.

[ref38] Herzog G.; Nolan M. T.; Arrigan D. W. M. Haemoglobin Unfolding Studies at the Liquid-Liquid Interface. Electrochem. Commun. 2011, 13, 723–725. 10.1016/j.elecom.2011.04.020.

[ref39] Poltorak L.; Herzog G.; Walcarius A. In-Situ Formation of Mesoporous Silica Films Controlled by Ion Transfer Voltammetry at the Polarized Liquid-Liquid Interface. Electrochem. Commun. 2013, 37, 76–79. 10.1016/j.elecom.2013.10.018.

[ref40] Poltorak L.; Herzog G. G.; Walcarius A. Electrochemically Assisted Generation of Silica Deposits Using a Surfactant Template at Liquid/Liquid Microinterfaces. Langmuir 2014, 30, 11453–11463. 10.1021/la501938g. 25229369

[ref41] Mareček V.; Jänchenová H. Electrochemically Controlled Formation of a Silicate Membrane at a Liquid|liquid Interface. J. Electroanal. Chem. 2003, 558, 119–123. 10.1016/S0022-0728(03)00386-3.

[ref42] Kickelbick G. Hybrid Materials, Synthesis, Characterization and Applications; Kickelbick G., Ed.; Wiley-VCH Verlag GmbH: Weinheim, Germany, 2007.

[ref43] Poltorak L.; Dossot M.; Herzog G.; Walcarius A. Interfacial Processes Studied by Coupling Electrochemistry at the Polarised Liquid–Liquid Interface with in Situ Confocal Raman Spectroscopy. Phys. Chem. Chem. Phys. 2014, 16, 26955–26962. 10.1039/C4CP03254C. 25377062

[ref44] Zhang X.; Wang J.; Wu W.; Liu C.; Qian S. Preparation of Amino-Functionalized Mesoporous Silica Thin Films with Highly Ordered Large Pore Structures. J. Sol-Gel Sci. Technol. 2007, 43, 305–311. 10.1007/s10971-007-1588-9.

[ref45] Sang L.-C.; Vinu A.; Coppens M.-O. General Description of the Adsorption of Proteins at Their Iso-Electric Point in Nanoporous Materials. Langmuir 2011, 27, 13828–13837. 10.1021/la202907f. 21958167

[ref46] Moerz S. T.; Huber P. Protein Adsorption into Mesopores: A Combination of Electrostatic Interaction, Counterion Release, and van Der Waals Forces. Langmuir 2014, 30, 2729–2737. 10.1021/la404947j. 24571263

[ref47] Vinu A.; Murugesan V.; Tangermann O.; Hartmann M. Adsorption of Cytochrome c on Mesoporous Molecular Sieves: Influence of PH, Pore Diameter, and Aluminum Incorporation. Chem. Mater. 2004, 16, 3056–3065. 10.1021/cm049718u.

[ref48] Hristova S.; Zhivkov A.; Atanasov B. Electrostatics of Horse Heart Cytochrome c and Montmorillonite Monolamellar Plate. Biotechnol. Biotechnol. Equip. 2009, 23, 568–571. 10.1080/13102818.2009.10818489.

[ref49] Hirota S.; Hattori Y.; Nagao S.; Taketa M.; Komori H.; Kamikubo H.; Wang Z.; Takahashi I.; Negi S.; Sugiura Y.; Kataoka M.; Higuchi Y. Cytochrome c Polymerization by Successive Domain Swapping at the C-Terminal Helix. Proc. Natl. Acad. Sci. U. S. A. 2010, 107, 12854–12859. 10.1073/pnas.1001839107. 20615990 PMC2919943

[ref50] Wang Z.; Ando Y.; Nugraheni A. D.; Ren C.; Nagao S.; Hirota S. Self-Oxidation of Cytochrome c at Methionine80 with Molecular Oxygen Induced by Cleavage of the Met-Heme Iron Bond. Mol. BioSyst. 2014, 10, 3130–3137. 10.1039/C4MB00285G. 25224641

[ref51] Zhao H. Z.; Du Q.; Li Z. S.; Yang Q. Z. Mechanisms for the Direct Electron Transfer of Cytochrome c Induced by Multi-Walled Carbon Nanotubes. Sensors 2012, 12, 10450–10462. 10.3390/s120810450. 23112609 PMC3472837

[ref52] Suemoto T.; Ebihara H.; Nakao H.; Nakajima M. Observation of Ultrafast Q-Band Fluorescence in Horse Heart Cytochrome c in Reduced and Oxidized Forms. J. Chem. Phys. 2011, 134, 034502 10.1063/1.3518370. 21261363

[ref53] Collinson M.; Bowden E. F. UV-Visible Spectroscopy of Adsorbed Cytochrome c on Tin Oxide Electrodes. Anal. Chem. 1992, 64, 1470–1476. 10.1021/ac00037a028.

[ref54] Liu H.; Tian Y.; Deng Z. Morphology-Dependent Electrochemistry and Electrocatalytical Activity of Cytochrome C. Langmuir 2007, 23, 9487–9494. 10.1021/la700817y. 17665934

[ref55] Cheng S. H.; Kao K. C.; Liao W. N.; Chen L. M.; Mou C. Y.; Lee C. H. Site-Specific Immobilization of Cytochrome c on Mesoporous Silica through Metal Affinity Adsorption to Enhance Activity and Stability. New J. Chem. 2011, 35, 1809–1816. 10.1039/c1nj20255c.

[ref56] Lee C. H.; Mou C. Y.; Ke S. C.; Lin T. S. Effect of Spin Configuration on the Reactivity of Cytochrome c Immobilized in Mesoporous Silica. Mol. Phys. 2006, 104, 1635–1641. 10.1080/00268970500501045.

[ref57] Lee C. H.; Lang J.; Yen C. W.; Shih P. C.; Lin T. S.; Mou C. Y. Enhancing Stability and Oxidation Activity of Cytochrome c by Immobilization in the Nanochannels of Mesoporous Aluminosilicates. J. Phys. Chem. B 2005, 109, 12277–12286. 10.1021/jp050535k. 16852515

[ref58] Kao K. C.; Lee C. H.; Lin T. S.; Mou C. Y. Cytochrome c Covalently Immobilized on Mesoporous Silicas as a Peroxidase: Orientation Effect. J. Mater. Chem. 2010, 20, 4653–4662. 10.1039/b925331a.

[ref59] Fedurco M.; Augustynski J.; Indiani C.; Smulevich G.; Antalík M.; Bánó M.; Sedlák E.; Glascock M. C.; Dawson J. H. The Heme Iron Coordination of Unfolded Ferric and Ferrous Cytochrome c in Neutral and Acidic Urea Solutions. Spectroscopic and Electrochemical Studies. Biochim. Biophys. Acta, Proteins Proteomics 2004, 1703, 31–41. 10.1016/j.bbapap.2004.09.013. 15588700

[ref60] Dave B. C.; Miller J. M.; Dunn B.; Valentine J. S.; Zink J. I. Encapsulation of Proteins in Bulk and Thin Film Sol-Gel Matrices. J. Sol-Gel Sci. Technol. 1997, 8, 629–634. 10.1007/BF02436913.

[ref61] Droghetti E.; Oellerich S.; Hildebrandt P.; Smulevich G. Heme Coordination States of Unfolded Ferrous Cytochrome C. Biophys. J. 2006, 91, 3022–3031. 10.1529/biophysj.105.079749. 16877519 PMC1578467

[ref62] Spiro T. G.; Strekas T. C. Resonance Raman Spectra of Heme Proteins. Effects of Oxidation and Spin State. J. Am. Chem. Soc. 1974, 96, 338–345. 10.1021/ja00809a004. 4361043

[ref63] Strekas T. C.; Spiro T. G. Cytochrome c: Resonance Raman Spectra. Biochim. Biophys. Acta, Protein Struct. 1972, 278, 188–192. 10.1016/0005-2795(72)90121-3. 4341727

[ref64] Döpner S.; Hildebrandt P.; Rosell F. I.; Mauk A. G. Alkaline Conformational Transitions of Ferricytochrome c Studied by Resonance Raman Spectroscopy. J. Am. Chem. Soc. 1998, 120, 11246–11255. 10.1021/ja9717572.

[ref65] Kitt J. P.; Bryce D. A.; Minteer S. D.; Harris J. M. Raman Spectroscopy Reveals Selective Interactions of Cytochrome c with Cardiolipin That Correlate with Membrane Permeability. J. Am. Chem. Soc. 2017, 139, 3851–3860. 10.1021/jacs.7b00238. 28221789

[ref66] Hu S.; Spiro T. G.; Morris I. K.; Singh J. P.; Smith K. M. Complete Assignment of Cytochrome c Resonance Raman Spectra via Enzymatic Reconstitution with Isotopically Labeled Hemes. J. Am. Chem. Soc. 1993, 115, 12446–12458. 10.1021/ja00079a028.

[ref67] Choi J.; Cho D. W.; Tojo S.; Fujitsuka M.; Majima T.; et al. Configurational Changes of Heme Followed of Cytochrome c Folding Reaction. Mol. BioSyst. 2015, 11, 218–222. 10.1039/C4MB00551A. 25358103

[ref68] Matos M. C. C.; Ilharco L. M. M.; Almeida R. M. M. The Evolution of TEOS to Silica Gel and Glass by Vibrational Spectroscopy. J. Non-Cryst. Solids 1992, 147–148, 232–237. 10.1016/S0022-3093(05)80622-2.

[ref69] Moore G. R.; Pettigrew G. W. Cytochromes c, Evolutionary, Structural and Physicochemical Aspects, 1st ed..; Springer-Verlag: Berlin, Heidelberg, 1990.

[ref70] Hlady V.; Buijs J.; Jennissen H. Methods for Studying Protein Adsorption. Methods Enzymol. 1999, 309, 402–429. 10.1016/S0076-6879(99)09028-X. 10507038 PMC2664293

[ref71] Haldar S.; Sil P.; Thangamuniyandi M.; Chattopadhyay K. Conversion of Amyloid Fibrils of Cytochrome c to Mature Nanorods through a Honeycomb Morphology. Langmuir 2015, 31, 4213–4223. 10.1021/la5029993. 25338286

[ref72] Fisher W. R.; Taniuchi H.; Anfinsen B. On the Role of Heme in the Formation of the Strucure of Cytochrome C. J. Biol. Chem. 1973, 248, 3188–3195. 10.1016/S0021-9258(19)44026-X. 4349479

[ref73] Weber G.; Teale F. J. W. Electronic Energy Transfer in Haem Proteins. Discuss. Faraday Soc. 1959, 27, 134–141. 10.1039/df9592700134.

[ref74] López-Bernabeu S.; Huerta F.; Morallón E.; Montilla F. Direct Electron Transfer to Cytochrome c Induced by a Conducting Polymer. J. Phys. Chem. C 2017, 121, 15870–15879. 10.1021/acs.jpcc.7b05204.

[ref75] Guillemin Y.; Etienne M.; Aubert E.; Walcarius A. Electrogeneration of Highly Methylated Mesoporous Silica Thin Films with Vertically-Aligned Mesochannels and Electrochemical Monitoring of Mass Transport Issues. J. Mater. Chem. 2010, 20, 6799–6807. 10.1039/c0jm00305k.

[ref76] Sillu D.; Kaushik Y.; Agnihotri S. Immobilization of Enzymes onto Silica-Based Nanomaterials for Bioprocess Applications. In Gels Horizons: From Science to Smart Materials; Tripathi A., Savio Melo J., Eds.; Springer: Singapore, 2021; p 666.

