# Supplementary material for: Electrogeneration of a Free-Standing Cytochrome c—Silica Matrix at a Soft Electrified Interface
Source: Langmuir. 2021 Mar 25;37(13):4033–41. doi: 10.1021/acs.langmuir.1c00409 (PMC8562870; doi:10.1021/acs.langmuir.1c00409)
Supplement: Supplementary file 1 — la1c00409_si_001.pdf [file la1c00409_si_001.pdf]

Supporting information

Electrogeneration of a free-standing Cytochrome *c* – silica matrix at a soft electrified interface

Alonso Gamero-Quijano,[1] Manuel Dossot,[2] Alain Walcarius,[2] Micheál D. Scanlon,[1]* Grégoire Herzog[2]*

[1] The Bernal Institute and Department of Chemical Sciences, School of Natural Sciences, University of Limerick (UL), Limerick V94 T9PX, Ireland

[2] Université de Lorraine, CNRS, LCPME, F-54000 Nancy, France

*Corresponding authors: micheal.scanlon@ul.ie; gregoire.herzog@univ-lorraine.fr

Supporting information table of contents

Experimental set-ups for electrochemistry and spectroelectrochemistry measurements 2

Control cyclic voltammetry of Cyt *c* in the absence of silica hydrogel 4

Protective effect of the silica on the 5

Electrochemical stabilisation of Cyt *c*@SiO_2 hydrogel after aqueous phase exchange 5

Experimental set-ups for electrochemistry and spectroelectrochemistry measurements

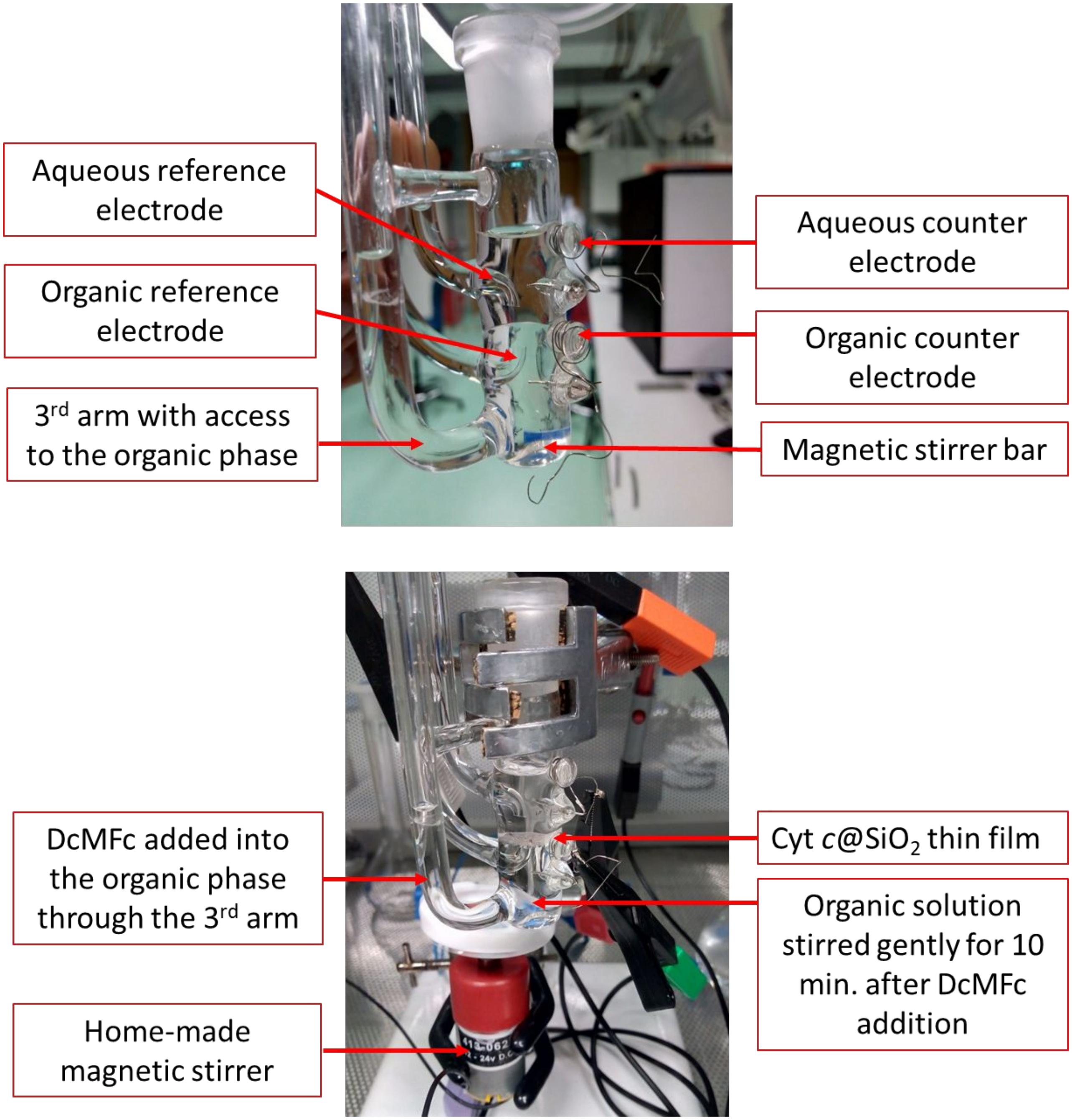

Figure S1. Homemade 4-electrode electrochemical cell with a third arm facilitating the addition of hydrophobic electron donors into the organic phase.

UV-vis spectroelectrochemistry setup

The spectrometer used was a USB 2000 Fiber Optic Spectrometer (Ocean Optics). The light source was a DH-2000-BAL deuterium–halogen (Ocean Optics) was guided through the optical fibre of 600 µm of diameter (Ocean Optics, USA). The light beam was collimated using optical lenses (Thor-lab, focal length: 2 cm) before and after the transmission of the beam through the electrochemical cell. The light beam passed through the electrochemical cell slightly above the water|TFT interface, i.e., through the aqueous phase. The interfacial Galvani potential difference ($\Delta_{\mathrm{o}}^{\mathrm{w}}\phi$) was controlled using an Autolab PGSTAT204 potentiostat (Metrohm, Switzerland). An image of this setup is shown in Figure S2.

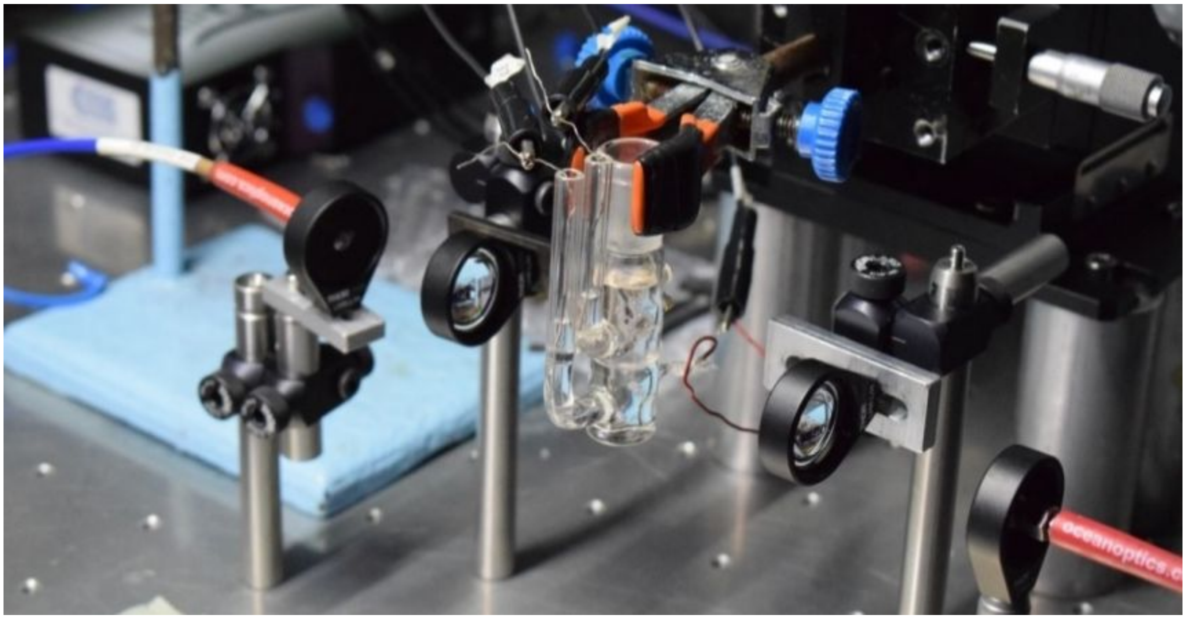

Figure S2. Optical setup for in situ parallel beam UV-vis absorbance measurements using a four-electrode electrochemical cell.

Control cyclic voltammetry of Cyt *c* in the absence of silica hydrogel

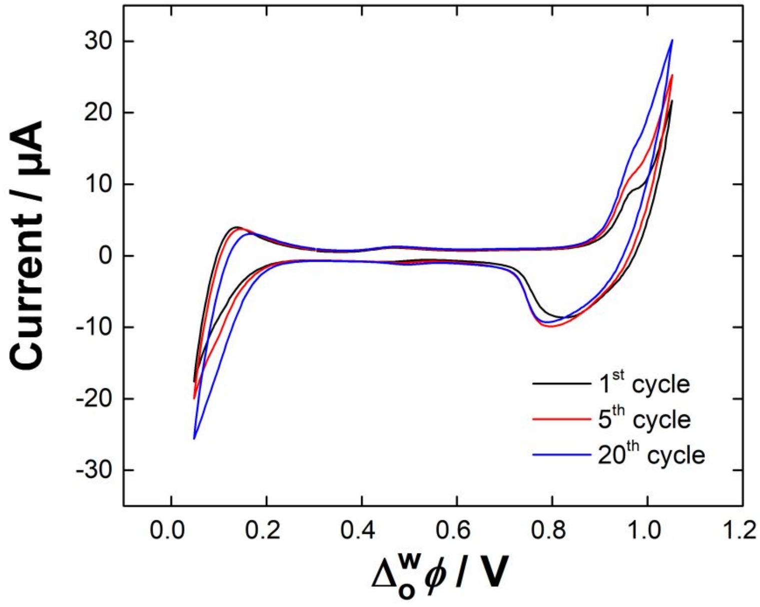

Figure S3. CV of 10 μM of Cyt *c* (pH 9) at a liquid-liquid interface in the absence of TEOS in the aqueous phase or CTA^+ in the organic phase. Aqueous phase: 5mM NaCl , Organic phase: 5mM BATB. ν = 20 mV s^{-1}.

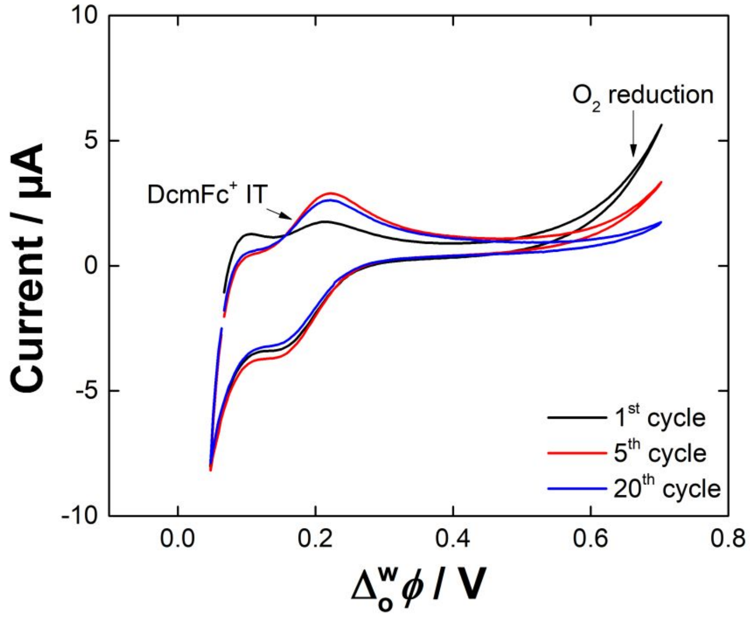

Figure S4. CV of 10 μM of Cyt c (aqueous phase) || 500 μM DcMFc (organic phase). ν = 20 mV s^{-1}.

Protective effect of the silica film

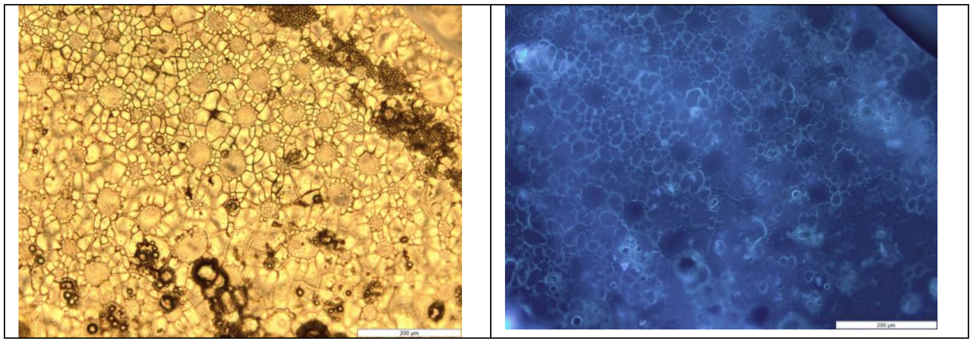

Figure S5. Optical and fluorescence micrographs of Cyt *c*@SiO_2 films, left: Cyt *c*@Silica film extracted from the ITIES and right: the same silica Cyt *c*@silica film after being immersed in 0.4 M NaCl solution for 2h, respectively.

Electrochemical stabilisation of Cyt *c*@SiO_2 hydrogel after aqueous phase exchange

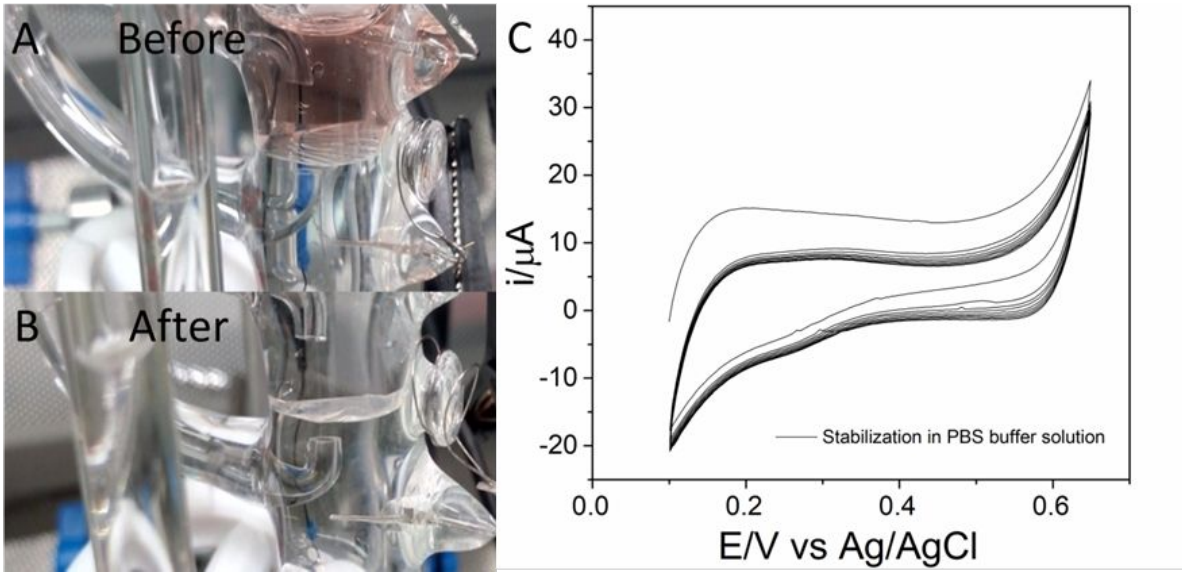

Figure S6. Image of the 4-electrode cell after (A) Cyt *c*@ SiO_2 electrogeneration and (B) after replacing the aqueous phase with 10 mM PBS solution. (C) Electrochemical stabilisation of Cyt *c*@SiO_2 by repetitive voltammetric scans at liquid-liquid interface (Electrochemical cell 3).
